# Mechanical and Metallurgical Properties of Various Nickel-Titanium Rotary Instruments

**DOI:** 10.1155/2017/4528601

**Published:** 2017-11-28

**Authors:** Kyu-Sang Shim, Soram Oh, KeeYeon Kum, Yu-Chan Kim, Kwang-Koo Jee, Seok Woo Chang

**Affiliations:** ^1^White Dental Clinic, Daemyeong Bld, 87 Choonggyeong-ro, Deokyang-gu, GoYang 10529, Republic of Korea; ^2^Department of Conservative Dentistry, Kyung Hee University Dental Hospital, 23 Kyungheedaero, Dongdaemun-gu, Seoul 02447, Republic of Korea; ^3^Department of Conservative Dentistry, Dental Research Institute, Seoul National University Dental Hospital and Seoul Dental Hospital for Disabled, Seoul National University School of Dentistry, 101 Daehawk-Ro, Seoul 03080, Republic of Korea; ^4^Biomedical Research Institute, Korea Institute of Science and Technology, 5 Hwarang-ro 14, Seongbuk-gu, Seoul 02792, Republic of Korea; ^5^Future Convergence Research Division, Korea Institute of Science and Technology, 5 Hwarang-ro 14, Seongbuk-gu, Seoul 02792, Republic of Korea; ^6^Department of Conservative Dentistry, School of Dentistry, Kyung Hee University, 23 Kyungheedaero, Dongdaemun-gu, Seoul 02447, Republic of Korea

## Abstract

The aim of this study was to investigate the effect of thermomechanical treatment on mechanical and metallurgical properties of nickel-titanium (NiTi) rotary instruments. Eight kinds of NiTi rotary instruments with sizes of ISO #25 were selected: ProFile, K3, and One Shape for the conventional alloy; ProTaper NEXT, Reciproc, and WaveOne for the M-wire alloy; HyFlex CM for the controlled memory- (CM-) wire; and TF for the R-phase alloy. Torsional fracture and cyclic fatigue fracture tests were performed. Products underwent a differential scanning calorimetry (DSC) analysis. The CM-wire and R-phase groups had the lowest elastic modulus, followed by the M-wire group. The maximum torque of the M-wire instrument was comparable to that of a conventional instrument, while those of the CM-wire and R-phase instruments were lower. The angular displacement at failure (ADF) for the CM-wire and R-phase instruments was higher than that of conventional instruments, and ADF of the M-wire instruments was lower. The cyclic fatigue resistance of the thermomechanically treated NiTi instruments was higher. DSC plots revealed that NiTi instruments made with the conventional alloy were primarily composed of austenite at room temperature; stable martensite and R-phase were found in thermomechanically treated instruments.

## 1. Introduction

Nickel-titanium (NiTi) rotary instruments have become essential tools in endodontics due to their superior flexibility and higher cutting efficiency compared to those of conventional stainless steel instruments [1]. Moreover, owing to superelasticity, NiTi instruments maintain the original canal shape with fewer irregularities, such as zip, ledge, or transportation during canal preparation [2]. Despite these advantages, NiTi instruments are not free from sudden file separation during treatment, which may lead to a poor prognosis [3].

Instrument separation during canal preparation can be broadly divided into two types: torsional fracture and cyclic fatigue fracture. Torsional fracture occurs when the instrument tip is tightly bound in the canal and the handpiece continues to rotate over the maximum strain it can withstand; cyclic fatigue fracture occurs when the instrument receives repeated stresses during cyclic rotation in a curved canal [3]. Research has included various attempts by manufacturers to overcome this drawback. These efforts include (i) modifying the design of the instrument, such as the cross-sectional form, taper, helical angle, and pitch length; (ii) changing the manufacturing process to use twisting instead of milling; and (iii) enhancing the surface of the instrument through special processes such as electropolishing [4, 5]. Recently, manufacturers have also focused their efforts not only on the modification of NiTi instruments as listed above, but also on improvement of the characteristics of the NiTi alloy [4, 6].

NiTi alloy has three different, temperature-dependent, microstructure phases: austenite, martensite, and R-phase [7]. Austenitic NiTi is strong and hard, while martensitic and R-phase NiTi are soft and ductile and can be easily deformed. The mechanical characteristics of NiTi are influenced by the compositions of the three phases [8]. The conventional NiTi alloy is primarily in the austenite phase at room temperature. Thermomechanical treatments could maintain the alloy in the martensite phase, R-phase, or mixed form by altering the transformation temperature and consequently changing the characteristics of the alloy [4, 7].

Several thermomechanically treated NiTi alloys have been released in recent years. M-wire (Dentsply Maillefer, Ballaigues, Switzerland) is reported to have higher flexibility than that of the conventional NiTi alloy [9]. The R-phase (Kerr Corp., Orange, CA, USA) NiTi alloy is another thermally treated NiTi alloy which is transformed from the austenite phase into the martensite phase during the manufacturing process [4]. CM-wire (DS Dental, Johnson City, TN, USA) is a novel NiTi alloy introduced in 2010. It has undergone a process that controls the memory of the material and makes the alloy extremely flexible; it does not rebound to its original shape like the conventional NiTi alloy [7]. The purpose of this study was to investigate and compare the mechanical and metallurgical characteristics of NiTi instruments made from different thermomechanically treated NiTi alloys.

## 2. Materials and Methods

### 2.1. NiTi Rotary Files

Eight NiTi instruments made from four different alloys were selected for this study. ProFile (Dentsply Maillefer), K3 (Kerr Corp.), and One Shape (Micro-Mega, Besanҫon, France) were made of conventional NiTi alloy (conventional group). ProTaper NEXT (Dentsply Maillefer), Reciproc (VDW GmbH, Munich, Germany), and WaveOne (Dentsply Maillefer) were made of M-wire (M-wire group). HyFlex CM (Coltène/Whaledent, Altstätten, Switzerland) was made of CM-wire (CM-wire group). Lastly, TF (Kerr Corp.) was made of R-phase (R-phase group). All products had an identical ISO #25 tip size and a length of 21 mm; the single exception was TF, which had a length of 23 mm. A constant taper of 0.06 was selected for the primary standard. However, it could not be completely matched through all instruments, as some had variable tapers or were not available. The detailed specifications of each instrument are listed in Table 1.

### 2.2. Torsional Fracture Test and Cyclic Fatigue Fracture Test

**Figure 1:**
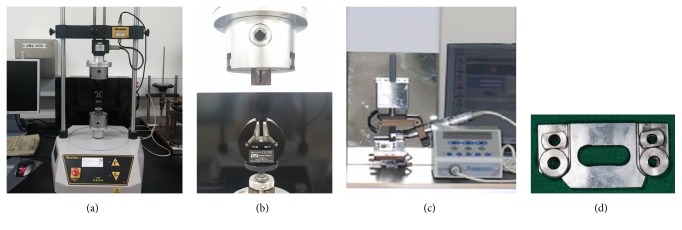
Fracture resistance testing devices used in this study. (a) The torsion testing machine (Vortex-i, Mecmesin Co., Slinfold, UK) and (b) the operating part from (a), magnified. The shaft is mounted on the upper part and the file tip is tightly bound at the lower part. (c) The cyclic fatigue tester (Denbotix, Bucheon, Korea), and (d) the artificial canal of the cyclic fatigue tester. The right part was used in this study.

Twelve unused instruments of each brand were used for the torsional fracture test. Every instrument was inspected visually for defects and deformities before the test. A torsion testing machine (Vortex-i, Mecmesin Co., Slinfold, UK) was used to evaluate the torsional resistance of the instrument (Figure 1(a)). The end of the shaft was clamped into a chuck connected to a reversible geared motor. Five millimeters of the instrument's tip was clamped in another chuck with brass jaws to prevent sliding. The Reciproc and WaveOne instruments were bound at a point 4 mm from the instrument's tip to compensate for the taper difference and to have equal diameter, as the diameter has been reported to influence torsional resistance [10]. A continuous clockwise rotation at a speed of 2 rpm was applied to the motor until failure occurred. Rotation was applied to the Reciproc and WaveOne instruments in a counterclockwise direction due to the opposite direction of the spiral flutes. The torsional torque value and angular displacement were continuously recorded until fracture using specifically designed computer program. The maximum torque and angular displacement at failure (ADF) were obtained. The torsion test curves were plotted from the torsional torque value and angular displacement by seconds. The elastic modulus (EM) was obtained by the slope on the linear part of the torsion test curve.

Twelve additional unused instruments of each brand underwent a cyclic fatigue fracture test using a fatigue tester (Denbotix, Bucheon, Korea) (Figure 1(c)). The tester was built as two components: an artificial canal and an arm. The artificial canal had a radius of 5 mm, an inner diameter of 1.5 mm, angle of curvature of 60 degrees, and length of 8 mm at the straight part and was made of stainless steel (Figure 1(d)). The arm, which was located above the artificial canal, was able to operate in an axial pecking motion while tightly holding the handpiece. The axial pecking motion was set to oscillate in a range of 6 mm at a speed of 0.5 cycles per second to simulate the clinical up-down motion. A 20 : 1 reduction handpiece with an electric torque-controlled motor (Aseptico, Woodinville, WA, USA) was fixed at the arm to mount the handle of each sample and to rotate it clockwise at the recommended rpm of each instrument (300 rpm for ProFile, K3, and ProTaper NEXT; 350 rpm for One Shape; 500 rpm for HyFlex CM and TF). The artificial canal was filled with RC-prep (Premier Dental Products, Norristown, PA, USA) during the test to minimize heat generation and friction against the canal wall. The time to fracture was recorded in seconds. The number of cycles to fracture (NCF) for each instrument was calculated by multiplying the time to fracture by the applied rpm.

Clinically, Reciproc and WaveOne are used with reciprocal movement. To investigate the effect of reciprocating motion on fatigue fracture resistance, cyclic fatigue fracture tests of Reciproc and WaveOne were conducted using both continuous and reciprocating rotation. A reciprocating handpiece and engine (VDW.SILVER RECIPROC, VDW GmbH) were connected to the fatigue tester. Twelve unused samples of Reciproc and WaveOne were subjected to cyclic fatigue testing with reciprocating rotation using the “RECIPROC ALL” mode for Reciproc and “WAVEONE ALL” mode for WaveOne. The manufacturer's information revealed that the “RECIPROC ALL” mode has a speed of 300 rpm and the “WAVEONE ALL” mode has a speed of 350 rpm [11, 12]. Twelve additional unused instruments of Reciproc and WaveOne were subjected to cyclic fatigue testing by continuous rotation: Reciproc at 300 rpm and WaveOne at 350 rpm. A 6-mm continuous up-and-down pecking motion was applied to cyclic fatigue testing in the reciprocating and continuous rotation modes. The time to fracture was recorded in seconds and the NCFs for both products were calculated by multiplying the time to fracture by the applied rpm.

After the torsional and cyclic fatigue fracture tests, the broken samples were cleaned with absolute alcohol for approximately 60 s. The surfaces were examined under a scanning electron microscope (SEM) (S-4700, Hitachi, Ltd., Tokyo, Japan) at various magnifications (×200–×5,000) to observe the fracture patterns.

EM, maximum torque, ADF, and NCF values were statistically analyzed by one-way ANOVA with SPSS Statistics for Windows, Version 22.0 (IBM, Armonk, NY, USA). A Tukey's HSD was performed for post hoc comparison. An independent sample* t*-test was used to compare the cyclic fatigue fracture results between rotational and reciprocal movements for Reciproc and WaveOne. Pearson correlation analyses between the EM and maximum torque and between the EM and NCF were conducted. A *p* value < 0.05 was considered significant.

### 2.3. DSC Analysis

The phase transformations of the NiTi alloy can be investigated by differential scanning calorimetry (DSC) analysis, in which the difference between the heat energy supplied and an inert specimen heated at the same rate is measured precisely. Three unused samples for each of the eight products were prepared for DSC analysis. The DSC analyses (Model 2920 DSC, TA Instruments, New Castle, DE, USA) were conducted over a temperature change starting at 25°C. Each instrument was heated to 100°C to obtain a heating curve and then cooled to −100°C to obtain a cooling curve. Finally, instruments were heated again to 80°C. The heating and cooling rates were 10°C/min. The heat flow of each specimen during the temperature change was recorded in diagram form, which was then analyzed by computer software (Advantage software, TA Instruments) to obtain the peak temperature points of the phase transformation and enthalpy changes associated with these processes.

## 3. Results

### 3.1. Torsional and Cyclic Fatigue Fracture Test Results

**Figure 2:**
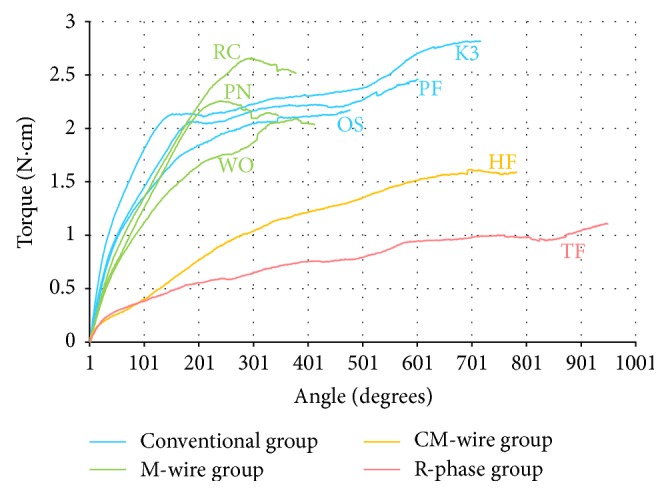
Representative torsion test curves of all products from the torsional fracture test. Note that each NiTi alloy group has a typical curve form. PF: ProFile, OS: One Shape, PN: ProTaper NEXT, RC: Reciproc, WO: WaveOne, and HF: HyFlex CM.

The representative torsion test curves obtained from the torsional fracture tests are plotted in Figure 2. The EM is defined as the slope of the linear part of the torsion test curve; a lower EM indicates greater flexibility. The mean EM, maximum torque, and ADF of each product are presented in Table 2. NiTi instruments made from CM-wire and R-phase had the lowest mean EMs, followed by the M-wire group (*p* < 0.05) and the conventional group. There was no significant difference in mean EM between the CM-wire and R-phase groups (*p* > 0.05).

The maximum torque is a torque level that a NiTi instrument can withstand while being twisted. There was no significant difference between the M-wire and conventional groups (*p* > 0.05); the CM-wire and R-phase groups had lower mean maximum torque than that of the conventional group (*p* < 0.05) (Table 2). The ADF is the amount of angular displacement of a NiTi instrument at failure. The CM-wire and R-phase groups had higher mean ADFs than that of the conventional group (*p* < 0.05) (Table 2). However, the mean ADF of the M-wire group was lower than those of ProFile, K3 instruments, which belonged to the conventional group (*p* < 0.05). This finding indicated that M-wire instruments were more likely to fracture than deform under a torsional stress. Additionally, regression analysis revealed a significant positive correlation between the EM and maximum torque (*p* < 0.01; Pearson *r* = 0.808). Less flexible (i.e., higher EM) instruments can withstand a higher maximum torque.

**Figure 3:**
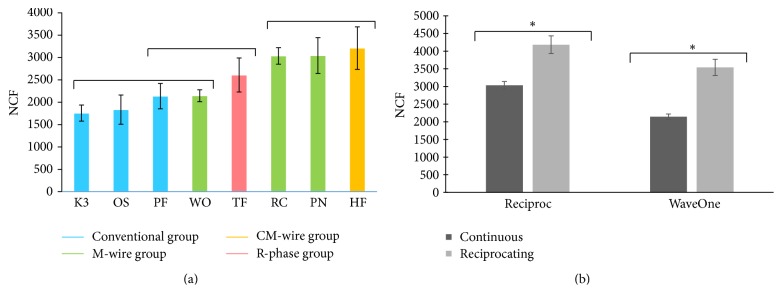
Results from the cyclic fatigue fracture test. (a) The mean number of cycles to fracture (NCF) as determined by the cyclic fatigue fracture test, in ascending order. Groups under the same bar did not show significant difference (*p* > 0.05). (b) The mean NCF as determined by the cyclic fatigue fracture test at continuous rotation and reciprocating rotation. An *∗* indicates a significant difference (*p* < 0.05). PF: ProFile, OS: One Shape, PN: ProTaper NEXT, RC: Reciproc, WO: WaveOne, and HF: HyFlex CM.

The mean NCF of each instrument is shown in Figure 3(a). HyFlex CM (CM-wire group) and Reciproc, ProTaper NEXT (M-wire) had the highest NCF (*p* < 0.05). All thermomechanical treated groups showed higher NCF than conventional group, except WaveOne which showed comparable NCF with conventional group. No significant correlation was observed between the EM and NCF groups (*p* > 0.05). The result of the cyclic fatigue test for Reciproc and WaveOne is shown in Figure 3(b). The mean NCF at reciprocating motion was higher than that of continuous motion for both Reciproc and WaveOne instruments (*p* < 0.05).

**Figure 4:**
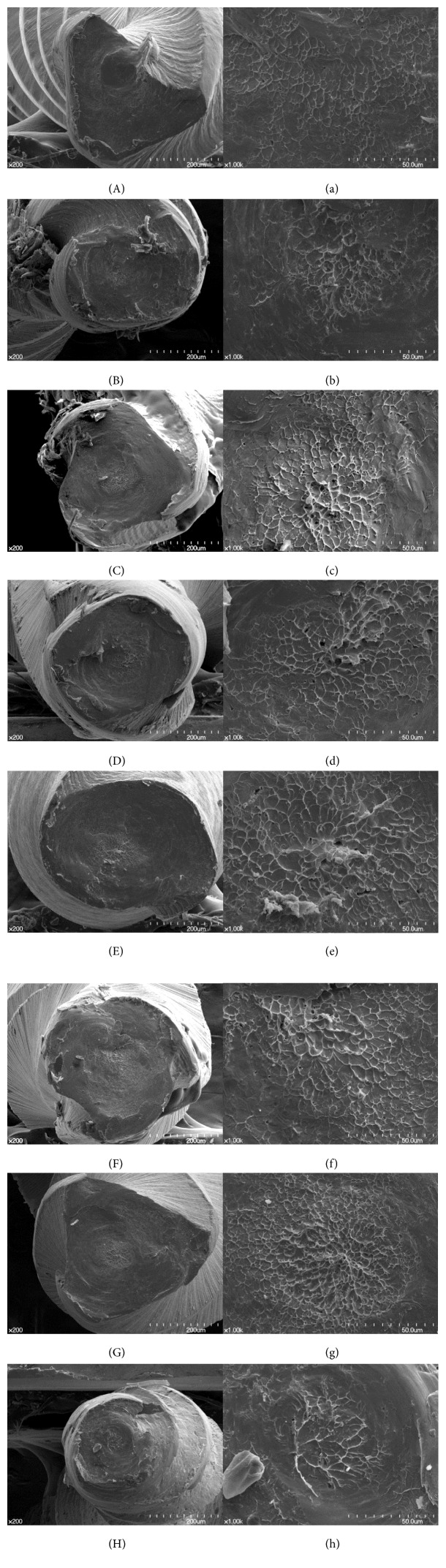
SEM images of the fractured surface of instruments after the torsional test: (A, a) ProFile, (B, b) K3, (C, c) One Shape, (D, d) ProTaper NEXT, (E, e) Reciproc, (F, f) WaveOne, (G, g) HyFlex CM, and (H, h) TF. All images at low magnification (A–H, ×200) show concentric circular abrasion marks and a “torn off” appearance. Skewed fibrous dimples near the center of rotation can be observed at a higher magnification (a–h, ×1,000).

**Figure 5:**
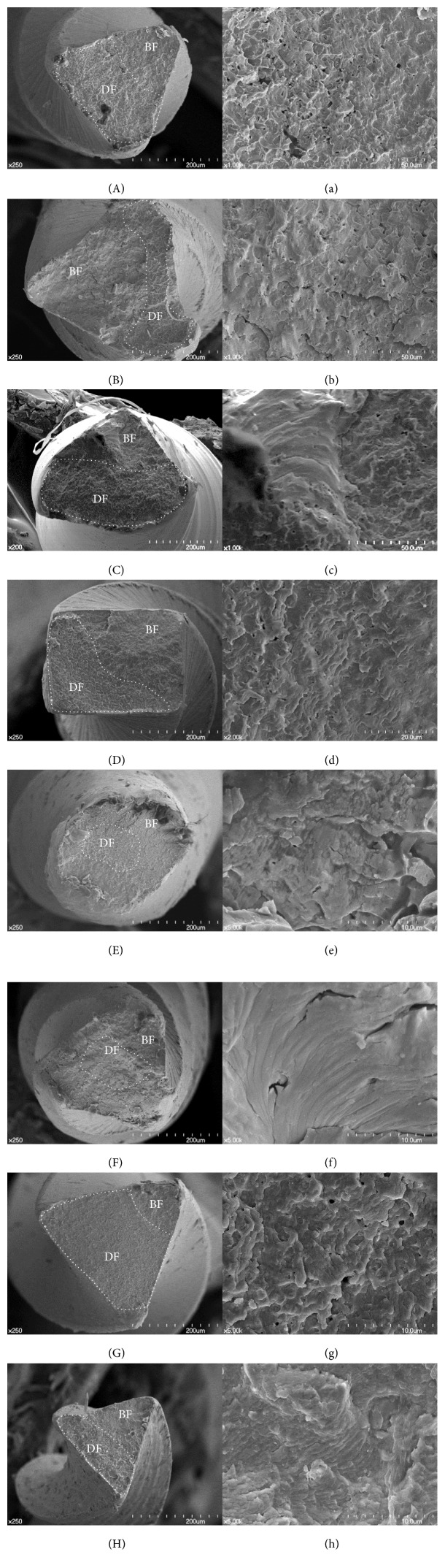
SEM images of the fractured instrument surfaces after a cyclic fatigue test: (A, a) ProFile, (B, b) K3, (C, c) One Shape, (D, d) ProTaper NEXT, (E, e) Reciproc, (F, f) WaveOne, (G, g) HyFlex CM, and (H, h) TF. All images at low magnification (A, B, D–H, ×250; C, ×200) show a mixed pattern of brittle fracture (BF) and ductile fracture (DF). The ductile fracture pattern with a dimple area is outlined with a dotted line. A brittle fracture pattern with fatigue striations can be observed more clearly in the higher magnification images (a–c, ×1,000; d, ×2,000; e–h, ×5,000).

SEM observation revealed typical surface patterns of torsional fracture, such as concentric circular abrasion marks and skewed fibrous dimples at the center of rotation from the torsional fracture test (Figure 4). The SEM images of fractured surfaces from the cyclic fatigue test also showed a typical pattern of fatigue fracture characterized by multiple striations (BF: brittle fracture) and clusters of dimples (DF: ductile fracture) (Figure 5).

### 3.2. DSC Analysis Results

**Figure 6:**
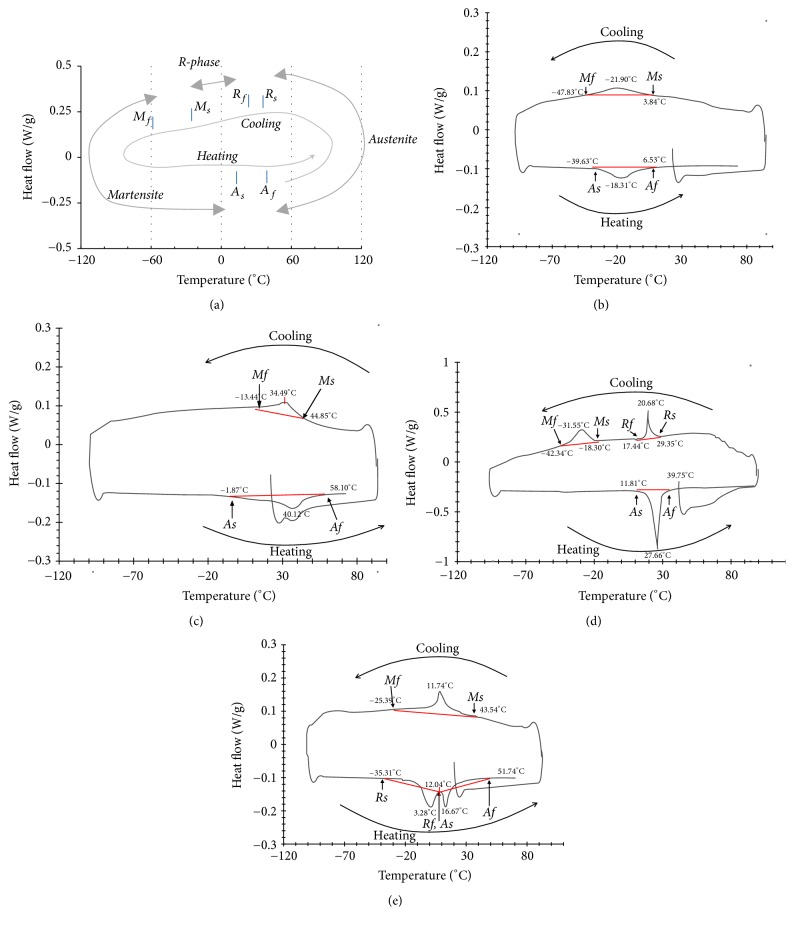
DSC plots, heating* (lower)* and cooling* (upper)* curves are shown. (a) A sample illustration of a DSC curve. *A*_*s*_ and *A*_*f*_ indicate the starting and finishing points of austenitic transformation in the heat curve, respectively. *M*_*s*_ and *M*_*f*_ indicate the start and end points of martensitic transformation in a cooling curve, respectively. *R*_*s*_ and *R*_*f*_ indicate the starting and finishing points of R-phase transformation in a heating or cooling curve, respectively. (b) DSC plot of K3, (c) DSC plot of Reciproc, (d) DSC plot of HyFlex CM, and (e) DSC plot of TF.

The DSC results are included in Figure 6. The results were recorded as a circular curve plotted in a counterclockwise direction. An example of DSC plot is depicted in Figure 6(a). The upper part denoted the cooling curve, and the lower part denoted the heating curve. Each part had several peaks. The phase transformations were observed through these endothermic peaks in the heating curve and exothermic peaks in the cooling curve, by which the phase of a NiTi alloy can be assumed at a given temperature. The starting point (*A*_*s*_) and finishing point (*A*_*f*_) of austenitic transformation were located in the heating curve; the starting point (*M*_*s*_) and finishing point (*M*_*f*_) of the martensitic transformation were located in the cooling curve. They were determined by the intersection of an extrapolated baseline and the maximum gradient line of the lambda-type DSC curve. An additional peak or combined peaks in the heating or cooling curves indicated R-phase; the starting point (*R*_*s*_) and finishing point (*R*_*f*_) were determined in the same manner. The enthalpy change (Δ*H*) of phase transformation was determined from the area surrounded by the DSC curve and the baseline. Although the DSC curves of all products differed from each other, the number of peaks and corresponding temperature ranges of identical NiTi alloy groups (conventional and M-wire alloy groups) were similar. The representative DSC plots of the four alloys are shown in Figures 6(b)–6(e). The specific values of *A*_*s*_, *A*_*f*_, *M*_*s*_, *M*_*f*_, *R*_*s*_, *R*_*f*_, and the corresponding Δ*H* are provided in Table 3.

The DSC curve of K3 (conventional alloy) exhibited a single endothermic peak on cooling and a single exothermic peak on heating. Both peaks were observed at a temperature below zero (−18.31°C at heating curve and −21.90°C at cooling curve) (Figure 6(b)). The DSC curve of Reciproc (M-wire) also showed single endothermic and exothermic peaks similar to the conventional group. However, the temperature of both peaks was increased to above room temperature (40.12°C at heating curve and 34.49°C at cooling curve) (Figure 6(c)). The DSC curve of HyFlex CM (CM-wire) presented two obviously separated exothermic peaks observed on the cooling curve. The first peak was located at room temperature (20.68°C); the following peak was located at a temperature far below zero (−31.55°C) (Figure 6(d)). The DSC curve of TF (R-phase alloy) demonstrating a pair of endothermic peaks was observed on the heating curve; the peaks were located close together around room temperature (3.28°C and 16.67°C) (Figure 6(e)).

## 4. Discussion

Superelasticity of the NiTi alloy is associated with the occurrence of martensitic transformation from austenite [7]. The application of stress causes the material to transform from austenite to martensite when a critical stress level is reached. Beyond this point any increase of deformation occurs without appreciable stress elevation, until the transformation is finished [7]. The soft, ductile characteristics of martensite play an important role in overcoming the fracture of an instrument [4]. Based upon this behavior and the properties of martensite, various thermomechanical treatments have been applied to the conventional NiTi alloy to maintain stable martensite and R-phase formations at room temperature [13]. These efforts were successful, as confirmed by this study.

The overall decrease of EM in all three thermomechanically treated groups can be paraphrased as an increase in the flexibility of the thermomechanically treated NiTi instruments. This result can be explained by several factors. First, it has been reported that thermomechanical treatment increased the austenitic transformation-finishing (*A*_*f*_) temperature [14]. That is in accordance with the DSC results of the present study, which revealed the *A*_*f*_ temperatures for thermomechanically treated group were above the room temperature (58.10°C for Reciproc, 39.75°C for HyFlex CM, and 51.74°C for TF). This implies the existence of stable martensite, which contributes increased flexibility, at room temperature [14]. Another factor that decreased the EM was the “training” effect of the superelastic behavior due to thermal cycling under stress, resulting in easier formation of the same type of martensite upon loading [15]. Therefore, the stress-induced martensitic transformation of this trained NiTi alloy occurred at a lower stress level, which consequently contributed to the improved flexibility of the instrument made from it. Lastly, the superior flexibility of thermomechanically treated NiTi alloy could be affected by “softening” of heat treatment associated with dislocation annealing and coalescence of precipitates [16]. Furthermore, the EMs of the CM-wire and R-phase groups were even lower than that of the M-wire group. The multiple exothermic peaks in the cooling curve of the CM-wire group and endothermic peaks in the heating curve of the R-phase group imply the existence of the R-phase at room temperature. The R-phase is an intermediate phase between austenite and martensite; it has a rhombohedral structure that can be formed during forward transformation from martensite to austenite on heating and during reverse transformation from austenite to martensite on cooling. Two overlapping endothermic peaks were observed on the heating curve of TF file, which means the presence of intermediate R-phase during transformation from martensite to austenite [17, 18]. The R-phase had a lower elastic modulus than those of martensite and austenite, and the transformation strain for R-phase transformation is less than one-tenth of that of martensitic transformation [8, 19]. Therefore, these characteristics of R-phase can be considered to be strong evidence for the superior flexibility of the CM-wire and R-phase groups.

Torsional fracture of a material occurs when two conditions occur simultaneously, such as stress exceeding the ultimate torsional strength and a twisting deformation exceeding the ADF. The former can be described using the term “mechanical resistance” and the latter by “ductility.” The maximum torque results indicate the highest torque that a file can withstand without failure. Although the CM-wire and R-phase groups showed significantly lower EM, they also demonstrated significantly lower maximum torque than that of the other groups. This inverse tendency between flexibility and maximum torque was confirmed by the moderate correlation between the EM and maximum torque of this study. In contrast, the M-wire group exhibited comparable mean maximum torque values to the conventional group. This result is also in agreement with previous studies where the Vickers micro-hardness of M-wire was as hard as conventional NiTi wire, resulting in similar torsional resistance between the two [20–22].

The ductility of a material can be described as the ability to be plastically deformed under stress without fracture, which was observed by the ADF values of the torsional fracture test. The ADFs of the CM-wire and R-phase groups were significantly higher than that of the conventional group. This improvement in plasticity of the CM-wire and R-phase groups may result from the existence of martensite and the R-phase, respectively, as shown in the DSC results. This could be because R-phase demonstrated reduced elastic modulus and bending moment [17, 18]. In addition, martensite, as opposed to austenite, was more likely to deform, as martensite has been reported to have a twinning process: internal lattice movement that does not break atomic bonds due to absorption of stresses [14]. The TF showed the highest ADF result numerically among all products. This result is not surprising, because unlike other instruments, the TF instrument was manufactured using a twisting process rather than grinding [18]. Therefore, the applied torque during the torsional fracture test, which was in the opposite direction from the initial twisting direction of manufacturing, returned the instrument to its original configuration by unwinding it [23]. An instrument that can tolerate a higher ADF will undergo a higher plastic deformation before breaking. This plastic deformation can be visualized when the instrument is withdrawn from the canal, which can provide a warning that torsional fracture is imminent. In this respect, the instruments of the CM-wire and R-phase groups have excellent safety factors. However, the ADF result of the M-wire group was lower than that of the conventional group, which agreed with previous studies [24, 25].

Thermomechanically treated NiTi instruments had higher NCFs than that of the conventional group. It has been reported that a NiTi alloy with a certain amount of martensite is more likely to have favorable fatigue resistance than a fully austenitic NiTi alloy, since the fatigue-crack growth resistance of martensite is superior to that of stable austenite [26]. Additionally, the phase transformation of martensite has an outstanding characteristic of damping stresses through energy resorption during its twinned phase structure. Therefore, the fatigue crack propagation speed of martensite is much slower than that of austenite [7]. The DSC results of the M-wire, CM-wire, and R-phase groups imply that the existence of martensite can be considered strong evidence for the superior cyclic fatigue resistance of thermomechanically treated NiTi instruments. HyFlex CM exhibited the lowest EM and highest NCF among all products, whereas the conventional group showed a higher EM and lower NCF than that of the other groups. Nevertheless, there was no significant correlation between EM and NCF. It is speculated that instrument design such as taper and cross-sectional configuration, in addition to alloy properties, affected the NCF [27].

According to the NCF results, cyclic fatigue resistance significantly improved on WaveOne and Reciproc by reciprocating motion, which concurs with previous studies [28–30]. The reciprocating motion consisted of repeated combinations of counterclockwise and clockwise rotation. Counterclockwise rotation is responsible for cutting, while clockwise rotation is used to periodically reduce stress. In this respect, the stress released by clockwise rotation can be regarded as a factor of the superior cyclic fatigue resistance of reciprocating motion, which increases the lifespan of the instrument [29]. In the present study, Reciproc had a longer cyclic fatigue life than that of WaveOne, which concurred with prior studies [11, 28, 31]. It has been reported that more flexible instrument had superior cyclic fatigue resistance [11, 31]. However, the EM of the two products was not significantly different in this study. Reciproc R was reported to have a smaller cross-sectional area than WaveOne Primary [11, 32]. The smaller cross-sectional area of the Reciproc R can explain the higher NCF.

In the present study, instrument designs such as cross-sectional configuration, pitch length, and variable taper were not standardized among instruments. Nevertheless, significant improvements in mechanical properties such as flexibility, resistance to cyclic fatigue fracture, and plasticity were observed for thermomechanically treated instruments. This enhancement of mechanical properties of NiTi instruments can be considered to be the effect of the composition of microstructures and modification of phase transformation behavior caused by thermomechanical treatments. As the microstructures and phase transformation behavior of the NiTi alloy are considered to have effects on mechanical properties, quantification of the amount of austenite, martensite, and R-phase in thermomechanically treated NiTi alloys is necessary in future studies.

It would be wise to select the appropriate NiTi instrument depending on the condition of the root canal. For instance, NiTi instruments with high maximum torque are suitable for a straight but calcified narrow canal; instruments with a high flexibility may be useful for a curved canal.

## 5. Conclusion

Instruments made from thermomechanical treated NiTi alloys had higher NCF and lower EM than the conventional NiTi wire files. In addition, NiTi rotary instruments made from CM-wire or R-phase alloy showed lower maximum torque and higher ADF than the conventional NiTi wire files. DSC plots revealed that the conventional NiTi instruments were primarily composed of austenite at room temperature, while stable martensite and R-phase were found in thermomechanically treated instruments.

## Figures and Tables

**Table 1:** Specifications of the NiTi instruments used in this study.

NiTi alloy	Product	Tip size	Taper	Length (mm)	Manufacturer
Conventional	ProFile	#25	Constant (0.06)	21	Dentsply Maillefer, Ballaigues, Switzerland
	K3	#25	Constant (0.06)	21	Kerr Corp., Orange, CA, USA
	One Shape	#25	Constant (0.06)	21	Micro-Mega, Besançon, France

M-wire	ProTaper NEXT	#25 (X2)	Variable	21	Dentsply Maillefer
	Reciproc	#25 (R25)	Variable	21	VDW GmbH, Munich, Germany
	WaveOne	#25 (Primary)	Variable	21	Dentsply Maillefer

CM-wire	HyFlex CM	#25	Constant (0.06)	21	Coltène/Whaledent, Altstätten, Switzerland

R-phase	TF	#25	Constant (0.06)	23	Kerr Corp.

**Table 2:** Mean values (standard deviation) of elastic modulus, maximum torque, and angular displacement at fracture from the torsional fracture test.

NiTi alloy	Conventional alloy			M-wire			CM wire	R-phase
Product	ProFile	K3	One Shape	ProTaper NEXT	Reciproc	WaveOne	HyFlex CM	TF
EM (N·cm/*θ*)	0.92 (0.14)^d^	0.72 (0.12)^c^	0.68 (0.08)^b,c^	0.53 (0.07)^b,c^	0.57 (0.06)^b,c^	0.50 (0.05)^b^	0.21 (0.04)^a^	0.23 (0.04)^a^
Maximum torque (N·cm)	2.54 (0.31)^c^	2.76 (0.3)^c^	2.16 (0.23)^b,c^	2.37 (0.21)^c^	2.7 (0.17)^c^	2.19 (0.22)^b,c^	1.66 (0.15)^a,b^	1.19 (0.18)^a^
ADF (°)	602 (83.4)^b,c^	711 (57.5)^c^	479 (40.7)^a,b^	347 (26.6)^a^	391 (37.4)^a^	414 (39.5)^a^	782 (47.8)^c^	951 (76.1)^d^

EM, elastic modulus; ADF, angular displacement at fracture. Different superscript letters in the same row indicate significant differences (*p* < 0.05).

**Table 3:** Transformation temperatures and associated energy from the DSC plots for different NiTi instruments.

	Cooling					
	*M*_*s*_ (°C)	*M*_*f*_ (°C)	Δ*H*_*M*_ (J/g)	*R*_*s*_ (°C)	*R*_*f*_ (°C)	Δ*H*_*R*_ (J/g)
K3	3.84	−47.83	2.786			
Reciproc	44.85	−13.44	2.576			
HyFlex CM	−18.30	−42.34	8.211	29.35	17.44	3.934
TF	43.54	−25.39	6.221			

	Heating					
	*A*_*s*_ (°C)	*A*_*f*_ (°C)	Δ*H*_*A*_ (J/g)	*R*_*s*_ (°C)	*R*_*f*_ (°C)	Δ*H*_*R*_ (J/g)

K3	−39.63	6.53	3.279			
Reciproc	−1.87	58.10	4.248			
HyFlex CM	11.81	39.75	19.07			
TF	12.04	51.74	0.183	−35.31	12.04	1.369

*M*_*s*_, martensitic transformation-starting temperature; *M*_*f*_, martensitic transformation-finishing temperature; Δ*H*_*M*_, enthalpy change of martensitic transformation; *R*_*s*_, R-phase transformation-starting temperature; *R*_*f*_, R-phase transformation-finishing temperature; Δ*H*_*R*_, enthalpy change of R-phase transformation; *A*_*s*_, austenitic transformation-starting temperature; *A*_*f*_, austenitic transformation-finishing temperature; Δ*H*_*A*_, enthalpy change of austenitic transformation.

## References

[1] Guelzow A., Stamm O., Martus P., Kielbassa A. M. (2005). Comparative study of six rotary nickel-titanium systems and hand instrumentation for root canal preparation. *International Endodontic Journal*.

[2] Liu S.-B., Fan B., Cheung G. S. P. (2006). Cleaning effectiveness and shaping ability of rotary ProTaper compared with rotary GT and manual K-Flexofile. *American Journal of Dentistry*.

[3] McGuigan M. B., Louca C., Duncan H. F. (2013). Endodontic instrument fracture: causes and prevention. *British Dental Journal*.

[4] Peters O. A., de Azevedo Bahia M. G., Pereira E. S. J. (2017). Contemporary root canal preparation: innovations in biomechanics. *Dental Clinics of North America*.

[5] Kaul R., Farooq R., Kaul V., Khateeb S. U., Purra A. R., Mahajan R. (2014). Comparative evaluation of physical surface changes and incidence of separation in rotary nickel-titanium instruments: An in vitro SEM study. *Iranian Endodontic Journal*.

[6] Choi J., Oh S., Kim Y.-C., Jee K.-K., Kum K., Chang S. (2016). Fracture resistance of K3 nickel-titanium files made from different thermal treatments. *Bioinorganic Chemistry and Applications*.

[7] Shen Y., Zhou H.-M., Zheng Y.-F., Peng B., Haapasalo M. (2013). Current challenges and concepts of the thermomechanical treatment of nickel-titanium instruments. *Journal of Endodontics*.

[8] Otsuka K., Wayman C. M. (1998). *Shape Memory Materials*.

[9] Pereira E. S. J., Gomes R. O., Leroy A. M. F. (2013). Mechanical behavior of M-Wire and conventional NiTi wire used to manufacture rotary endodontic instruments. *Dental Materials*.

[10] Melo M. C. C., Pereira E. S. J., Viana A. C. D., Fonseca A. M. A., Buono V. T. L., Bahia M. G. A. (2008). Dimensional characterization and mechanical behaviour of K3 rotary instruments. *International Endodontic Journal*.

[11] Kim H.-C., Kwak S.-W., Cheung G. S.-P., Ko D.-H., Chung S.-M., Lee W. (2012). Cyclic fatigue and torsional resistance of two new nickel-titanium instruments used in reciprocation motion: reciproc versus WaveOne. *Journal of Endodontics*.

[12] Fidler A. (2014). Kinematics of 2 reciprocating endodontic motors: the difference between actual and set values. *Journal of Endodontics*.

[13] Alapati S. B., Brantley W. A., Iijima M. (2009). Micro-XRD and temperature-modulated DSC investigation of nickel-titanium rotary endodontic instruments. *Dental Materials*.

[14] Shen Y., Zhou H.-M., Zheng Y.-F., Campbell L., Peng B., Haapasalo M. (2011). Metallurgical characterization of controlled memory wire nickel-titanium rotary instruments. *Journal of Endodontics*.

[15] Otsuka K., Ren X. (2005). Physical metallurgy of Ti-Ni-based shape memory alloys. *Progress in Materials Science*.

[16] Chang S. W., Kim Y.-C., Chang H. (2013). Effect of heat treatment on cyclic fatigue resistance, thermal behavior and microstructures of K3 NiTi rotary instruments. *Acta Odontologica Scandinavica*.

[17] Hou X. M., Yahata Y., Hayashi Y., Ebihara A., Hanawa T., Suda H. (2011). Phase transformation behaviour and bending property of twisted nickel-titanium endodontic instruments. *International Endodontic Journal*.

[18] Braga L. C., Magalhães R. R. S., Nakagawa R. K. L., Puente C. G., Buono V. T. L., Bahia M. G. A. (2013). Physical and mechanical properties of twisted or ground nickel-titanium instruments. *International Endodontic Journal*.

[19] Wu S. K., Lin H. C., Chou T. S. (1990). A study of electrical resistivity, internal friction and shear modulus on an aged Ti49Ni51 alloy. *Acta Metallurgica et Materialia*.

[20] Pereira E. S. J., Peixoto I. F. C., Viana A. C. D. (2012). Physical and mechanical properties of a thermomechanically treated NiTi wire used in the manufacture of rotary endodontic instruments. *International Endodontic Journal*.

[21] Johnson E., Lloyd A., Kuttler S., Namerow K. (2008). Comparison between a novel nickel-titanium alloy and 508 nitinol on the cyclic fatigue life of ProFile 25/.04 rotary instruments. *Journal of Endodontics*.

[22] Ye J., Gao Y. (2012). Metallurgical characterization of M-Wire nickel-titanium shape memory alloy used for endodontic rotary instruments during low-cycle fatigue. *Journal of Endodontics*.

[23] Wycoff R. C., Berzins D. W. (2012). An *in vitro* comparison of torsional stress properties of three different rotary nickel-titanium files with a similar cross-sectional design. *Journal of Endodontics*.

[24] Kramkowski T. R., Bahcall J. (2009). An In Vitro Comparison of Torsional Stress and Cyclic Fatigue Resistance of ProFile GT and ProFile GT Series X Rotary Nickel-Titanium Files. *Journal of Endodontics*.

[25] Ninan E., Berzins D. W. (2013). Torsion and bending properties of shape memory and superelastic nickel-titanium rotary instruments. *Journal of Endodontics*.

[26] McKelvey A. L., Ritchie R. O. (1999). Fatigue-crack propagation in Nitinol, a shape-memory and superelastic endovascular stent material. *Journal of Biomedical Materials Research Part B: Applied Biomaterials*.

[27] Tripi T. R., Bonaccorso A., Condorelli G. G. (2006). Cyclic fatigue of different nickel-titanium endodontic rotary instruments. *Oral Surgery, Oral Medicine, Oral Pathology, Oral Radiology, and Endodontology*.

[28] Pedullà E., Grande N. M., Plotino G., Gambarini G., Rapisarda E. (2013). Influence of continuous or reciprocating motion on cyclic fatigue resistance of 4 different nickel-titanium rotary instruments. *Journal of Endodontics*.

[29] De-Deus G., Moreira E. J. L., Lopes H. P., Elias C. N. (2010). Extended cyclic fatigue life of F2 ProTaper instruments used in reciprocating movement. *International Endodontic Journal*.

[30] Varghese N., Pillai R., Sujathen U., Sainudeen S., Antony A., Paul S. (2016). Resistance to torsional failure and cyclic fatigue resistance of ProTaper Next, WaveOne, and Mtwo files in continuous and reciprocating motion: An *In Vitro* Study. *Journal of Conservative Dentistry*.

[31] Dagna A., Poggio C., Beltrami R., Colombo M., Chiesa M., Bianchi S. (2014). Cyclic fatigue resistance of OneShape, Reciproc, and WaveOne: An in vitro comparative study. *Journal of Conservative Dentistry*.

[32] de Magalhães R. R. S., Braga L. C. M., Pereira É. S. J., Peixoto I. F. D. C., Buono V. T. L., Bahia M. G. D. A. (2016). The impact of clinical use on the torsional behavior of Reciproc and WaveOne instruments. *Journal of Applied Oral Science*.

